# Biofilm Formation and Its Relationship with the Microbiome in Pediatric Otitis Media

**DOI:** 10.3390/microorganisms13122760

**Published:** 2025-12-04

**Authors:** Ana Jotic, Ivana Cirkovic, Nevena Jovicic, Bojana Bukurov, Natalija Krca, Katarina Savic Vujovic

**Affiliations:** 1 Clinic for Otorhinolaryngology and Maxillofacial Surgery, University Clinical Center of Serbia, Pasterova 2, 11000 Belgrade, Serbia; anajotic@yahoo.com (A.J.); bojanabukurov@gmail.com (B.B.); 2 Faculty of Medicine, University of Belgrade, Dr Subotica 4, 11000 Belgrade, Serbia; jovicic.nevena@gmail.com; 3 Institute of Microbiology and Immunology, Faculty of Medicine, University of Belgrade, Dr Subotica 1, 11000 Belgrade, Serbia; natalijakrca@gmail.com; 4 Faculty of Medicine, University Children’s Clinic, Tirsova 10, 11000 Belgrade, Serbia; 5 Department of Pharmacology, Clinical Pharmacology and Toxicology, Faculty of Medicine, University of Belgrade, Dr Subotica 1, 11000 Belgrade, Serbia; katarinasavicvujovic@gmail.com

**Keywords:** pediatric otitis media, middle ear microbiome, biofilm formation, microbiota dysbiosis, antibiofilm therapy

## Abstract

Otitis media is among the most common pediatric illnesses globally, constituting a leading cause of antimicrobial prescriptions, recurrent medical consultations, and preventable hearing loss in early childhood. Traditionally regarded as a sterile cavity intermittently invaded by pathogens, the middle ear is now recognized as a dynamic ecological niche influenced by anatomical immaturity of the Eustachian tube, host immune development, and the composition of resident microbial communities. Increasing evidence demonstrates that microbial dysbiosis and the establishment of biofilms are central to the persistence and recurrence of disease. This review synthesizes current knowledge of the pediatric middle ear microbiome, highlighting how commensal organisms contribute to mucosal resilience and colonization resistance, whereas pathogenic bacteria exploit ecological disruption to establish biofilm communities. Biofilm formation provides bacteria with enhanced survival through immune evasion, altered microenvironments, and antibiotic tolerance, thereby transforming acute otitis media into recurrent or chronic states. Furthermore, studies demonstrate how adenoids act as reservoirs of biofilm-forming organisms, seeding the middle ear and perpetuating infection. The emerging ecological perspective emphasizes the limitations of conventional antibiotic-centered management and directs attention toward innovative strategies, including microbiome-preserving interventions, probiotic or live biotherapeutic approaches, and antibiofilm agents. By defining pediatric otitis media as a disorder of disrupted host–microbe equilibrium, future research may pave the way for precision-based preventive and therapeutic strategies aimed at reducing the global burden of this pervasive disease.

## 1. Introduction

Otitis media (OM) is one of the most common pediatric diseases worldwide, accounting for millions of healthcare visits and antibiotic prescriptions annually. By the age of three, nearly 80% of children will experience at least one episode of acute otitis media (AOM), while around 40% will have three or more recurrent episodes [1,2]. Chronic otitis media with effusion (COME) affects up to 20% of children and can lead to conductive hearing loss, speech delay, impaired language development, and reduced quality of life [3]. The incidence of OM peaks between 6 months and 2 years of age, a period that coincides with both anatomical and immunological vulnerabilities specifically, the presence of short, more horizontal Eustachian tubes, and an immature immune system that is still developing its full protective capacity [4]. Although the prevalence decreases with age, older children with comorbidities such as allergic rhinitis or craniofacial abnormalities remain at risk [5]. The overall socioeconomic impact of OM is substantial, with an estimated global incidence of more than 700 million new cases annually, including approximately 50% occurring in children under five years of age. The disease contributes to over 30 million disability-adjusted life years (DALYs) lost each year, accounting for considerable healthcare costs, parental work absenteeism, and its role in driving antimicrobial resistance [6].

Epidemiology varies by region. In high-income countries, widespread use of *Streptococcus pneumoniae* conjugate vaccines and *Haemophilus influenzae* type b vaccines have reduced severe OM and related complications. However, the serotype replacement and persistence of nontypeable *H. influenzae* (NTHi) and *Moraxella catarrhalis* continue to maintain a significant disease burden [7,8]. In low- and middle-income countries, OM remains a major cause of morbidity and mortality due to limited vaccination coverage, poor healthcare access, overcrowding, and higher prevalence of respiratory infections. Chronic suppurative otitis media (CSOM) and hearing loss remain major challenges in these regions [9].

Risk factors for OM include prematurity, low birth weight, genetic predisposition, immunodeficiency, exposure to tobacco smoke, daycare attendance, lack of breastfeeding, and socioeconomic disadvantage [10]. Microbiological factors are also central, as colonization by *S. pneumoniae*, NTHi, and *M. catarrhalis* is strongly linked to recurrence and chronicity [11,12].

Traditionally, the middle ear was considered sterile, with pathogens invading only during episodes of acute infection. However, molecular approaches have demonstrated that the middle ear harbors a resident microbiome with diverse bacterial communities [13]. This paradigm shift has redefined OM as a disease resulting from complex interactions among microbes, host immunity, and environmental factors, rather than solely from pathogen invasion [14]. These insights underscore the importance of studying the microbiome to gain a deeper understanding of OM pathogenesis and to develop novel preventive and therapeutic strategies.

The aim of this review was to examine the interaction between biofilm formation and the middle ear microbiome in pediatric otitis media. By exploring how microbiome dysbiosis promotes biofilm development, and how biofilms reshape microbiota composition, we can highlight their mutual significance in the pathogenesis and persistence of disease.

## 2. Materials and Methods

This review was conducted as a targeted narrative literature analysis aimed at synthesizing current and historical evidence on the relationship between the middle ear microbiome, biofilm formation, and therapeutic approaches in pediatric OM. A systematic search was performed exclusively in the PubMed/MEDLINE database using the following Boolean string: (“otitis media” [MeSH Terms] OR “middle ear infection” OR “chronic otitis media” OR “otitis media with effusion”) AND (“microbiome” OR “microbiota” OR “dysbiosis”) AND (“biofilm” OR “biofilm formation” OR “quorum sensing”) AND (“child” OR “pediatric” OR “infant”) AND (“therapy” OR “antibiotic” OR “probiotic” OR “antibiofilm” OR “microbiome modulation”). Filters were applied to include human studies, English-language publications, and pediatric populations aged 18 years or younger. The last search was conducted on 25 October 2025, covering the time window from 2000 to 2025, which corresponds to the era of molecular and sequencing-based microbiome research in OM. Earlier seminal publications predating 2000 were also included when considered methodologically or conceptually relevant, such as foundational studies on middle ear anatomy, Eustachian tube physiology, or early biofilm biology.

Titles and abstracts were manually screened for eligibility, followed by full-text evaluation. Studies were included if they reported microbiological, molecular, immunological, or clinical data related to the microbiome, dysbiosis, or biofilm formation in pediatric OM, including acute, recurrent, chronic, or effusion forms. Both clinical and experimental designs were considered, encompassing sequencing-based microbiome analyses (16S rRNA, metagenomics, metatranscriptomics), in vitro and translational biofilm models, and systematic or narrative reviews relevant to the topic. Publications exploring therapeutic or preventive interventions, including antibiotics, probiotics, live biotherapeutics, or antibiofilm agents, were also included. Studies were excluded if they were conducted exclusively in adults, relied solely on animal or computational models without pediatric or translational relevance, or were unrelated to the middle ear or nasopharyngeal microbiota. Non-peer-reviewed materials, preprints, and conference abstracts without full text were not considered.

Given the heterogeneity of study designs, data synthesis followed a narrative evidence-weighting approach. Greater interpretive weight was assigned to studies employing high-resolution molecular techniques and clinical pediatric populations. The overall consistency and reliability of the data were assessed qualitatively using modified GRADE principles, with emphasis on methodological rigor, reproducibility, and direct applicability to pediatric otitis media.

## 3. Anatomy and Immunology of the Pediatric Middle Ear

### 3.1. Anatomical Features of the Pediatric Eustachian Tube

The Eustachian tube (ET) is a complex fibrocartilaginous organ that plays a crucial homeostatic role in the middle ear by providing pressure equalization, draining secretions, and serving as a mechanical barrier against nasopharyngeal pathogens. In infants and young children, anatomical immaturities combined with underdeveloped immune and mucociliary defense systems result in less effective ET function. Insufficient ventilation and secretion clearance from the middle ear contribute significantly to the high incidence of recurrent acute otitis media (RAOM) and COME.

At birth and during early childhood, the ET is shorter and more horizontal than in adults, measuring around 16–18 mm (vs. ~30–38 mm in adults), with an angle of ~10° relative to the skull base (~35–45° in adults) [15,16]. The pediatric ET is also wider at the distal end, more flaccid, and lacks full cartilage support and elastin in the hinge/recoil regions (its cartilaginous portion is relatively more compliant and distensible in children) [17]. Furthermore, the tensor veli palatini muscle, which is the main active opener of the cartilaginous ET, has a smaller cross-section, a less favorable vector of pull, and shorter attachment to the ET cartilage in infants [18].

These anatomical differences lead to increased reflux of nasopharyngeal secretions due to the low angle and immature valve mechanisms at the nasopharyngeal opening, impaired drainage and poor aeration of the middle ear, and easier entry of pathogens from the nasopharynx. Because the ET cannot open reliably or fully, negative middle ear pressure develops, effusion accumulates, and infections more easily establish. Anatomical immaturity correlates with epidemiologic data: the peak incidence of OM (both AOT and COME) occurs between 6 and 24 months of age, when ET anatomy and associated musculature are still immature.

### 3.2. Local Immune Defense Mechanisms of the Middle Ear

The immune defenses of the middle ear are underdeveloped in early childhood and gradually mature over the first few years of life. Newborns and infants have lower levels of immunoglobulins (especially IgA), reduced neutrophil function (e.g., oxidative burst), and less effective epithelial immune responses. Adaptive immune components build up with antigen exposure over time (while specific quantitative data for all these items are sparse in the literature, the trend is well documented) [19]. The mucosal epithelial cells of the ET and middle ear express pattern recognition receptors (like Toll-like receptors, TLRs) and secrete cytokines/chemokines (IL-1β, TNF-α, IL-8), which recruit neutrophils and monocytes. In infants, TLR responsiveness is lessened, delaying or reducing expression [20]. Over time, middle ear mucosa and associated lymphoid tissue (MALT, including the adenoids) contribute to the production of IgA in secretions, which helps prevent bacterial adherence. Secretory IgA has been found in middle ear effusions (MEE) and shares antigen specificities with nasopharyngeal secretions, reflecting similar mucosal immunity in the upper airway [21]. Secondary adaptive immune response is mediated by memory T and B cells, which produce high-affinity IgG into serum and local tissues. Comparisons between AOT and COME show differing immune profiles. Acute cases tend to have stronger pro-inflammatory cytokine responses and immune cell recruitment, whereas chronic effusions are more characterized by persistent lower-grade inflammation and sometimes suppressive features, such as increased levels of regulatory cytokines (e.g., IL-10, TGF-β) and the presence of regulatory T cells that dampen local immune activity [20,22].

The middle ear and ET are lined with a ciliated pseudostratified epithelium similar to respiratory mucosa. Goblet cells secrete mucus that traps microbes, and ciliary beating transports that mucus out toward the nasopharynx while tight junctions between epithelial cells maintain barrier integrity, preventing paracellular pathogen entry. However, in children, ciliary ultrastructure and function may not yet be fully mature, and viral infections (e.g., RSV) can damage cilia, impairing mucociliary transport. In addition, inflammatory conditions (infection, allergy) further disrupt the airway epithelial barrier by impairing ciliary function and breaking down tight junctions. Cytokines, like IL-4, IL-5, and IL-13, along with allergen-activated proteases, decrease the expression of tight junction proteins, leading to increased permeability and loss of ciliated cells. This damage, combined with reduced ciliary beat frequency and abnormal ciliary distribution, prevents effective mucus clearance, allowing pathogens and allergens to penetrate deeper into the airway [23]. Children with congenital or genetic conditions such as cleft palate, Down syndrome, or primary ciliary dyskinesia have particularly compromised mucociliary function and a higher risk of COME/RAOM.

### 3.3. Clinical Implications

**Figure 1:**
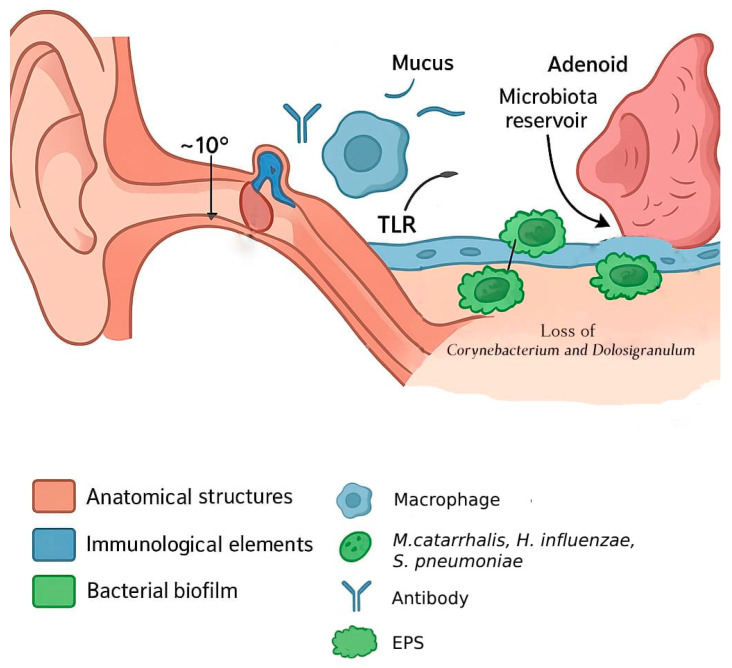
Anatomy and immunology of the pediatric middle ear with potential biofilm formation sites. The schematic depicts anatomical and immunological features of the pediatric middle ear relevant to OM pathogenesis. The shorter and more horizontal Eustachian tube (~10°) is predisposed to reflux of nasopharyngeal secretions and impaired aeration. The adenoid serves as a microbiota reservoir, where the loss of commensals enables colonization by otopathogens. Biofilms form on the mucosal surface, protected by an EPS, while immune elements such as macrophages, antibodies, and TLRs provide partial mucosal defense during early immune maturation.

The combination of anatomical immaturity (shorter, more horizontal ET with weak or inefficient active opening), underdeveloped innate and adaptive immunity, and less efficient mucociliary clearance predisposes infants and toddlers to negative middle ear pressure, effusions, and recurrent infections. RAOM and COME epidemiologic peak incidence at 6–24 months coincides with maximal anatomical and immunological immaturity of ET. As the tube elongates, the angle becomes more vertical, the cartilage stiffens, and the tensor veli palatini muscle becomes more functional, allowing for more effective ventilation and clearance [24]. The maturation of mucosal and systemic immune defenses, including enhanced secretory IgA production and improved innate signaling and mucociliary function, contributes to greater resistance to infection [25]. Together, these developmental changes reduce the likelihood of middle ear effusion, pathogen entry, and inflammation, explaining the sharp decline in otitis media incidence after early childhood (Figure 1).

Alterations in these host defense mechanisms are reflected in shifts within the microbial communities colonizing the nasopharynx and middle ear, linking the developmental anatomy and immunity of early childhood with subsequent microbiome composition and disease susceptibility.

## 4. The Middle Ear Microbiome: Commensals and Pathogens

The human middle ear is increasingly recognized as a dynamic ecological niche with a diverse and adaptable microbial community. This microbiome plays a dual role: certain bacteria function as commensals that contribute to mucosal homeostasis, while classical otopathogens can disrupt this balance and initiate disease processes such as AOM, COME, and CSOM [26,27].

Recent advances in molecular methods, particularly 16S rRNA amplicon sequencing, have revealed that the middle ear has a richer and more diverse bacterial community than traditional culture techniques had suggested [28,29]. Studies demonstrate that healthy ears or less disease-prone individuals are often colonized by commensal bacteria such as *Corynebacterium* spp. and *Dolosigranulum* spp., whereas diseased ears are dominated by known otopathogens (*H. influenzae*, *S. pneumoniae*, *M. catarrhalis*) or by less common bacteria such as *Alloiococcus otitidis* and *Turicella otitidis* [30,31,32].

Importantly, the balance between commensals and pathogens influences not only the onset of infection but the structure of biofilms, the resilience of the microbial ecosystem, and clinical outcomes. Understanding this interplay is central to developing microbiome-targeted interventions and alternatives to traditional antibiotic strategies [33].

### 4.1. Characterization of the Healthy Middle Ear Microbiota

Subsequent sequencing-based investigations have provided detailed characterization of the bacterial taxa present in the middle ear mucosa and effusions. Minami et al. profiled 155 middle ear samples and found that normal middle ears, particularly in children, were dominated by *Proteobacteria*, with contributions from *Actinobacteria*, *Firmicutes*, and *Bacteroidetes* [28]. Microbial composition differed significantly between children and adults, suggesting an age-dependent development of the middle ear microbiome.

Liu et al. used 16S rRNA pyrosequencing to analyze a pediatric case with chronic serous OM and compared the middle ear with adenoid and tonsil tissue. They detected *Pseudomonadaceae* as the dominant family in the middle ear, while the adenoid had a more diverse community overlapping with both tonsillar and middle ear microbiota [26]. This finding supports the concept of the adenoids as a microbial reservoir, with potential to induce both middle ear and tonsillar infections.

More recent comparative studies, such as those by Chan et al. and Jervis-Bardy et al., have reinforced that the “healthy” or less-diseased middle ear tends to exhibit greater microbial diversity and often includes bacteria traditionally regarded as commensals or skin-associated microbiota, such as *Corynebacterium* spp., coagulase-negative *Staphylococcus*, and *Cutibacterium* spp. [29,30]. These microorganisms are thought to play neutral or even beneficial roles in maintaining microbial balance, contrasting with the dominance of *H. influenzae*, *S. pneumoniae*, or *M. catarrhalis* observed in acute or recurrent infections.

The current evidence suggests that the healthy middle ear microbiota is a low-biomass, age-dependent community enriched with commensal bacteria, which may contribute to immune tolerance and barrier maintenance. Understanding its baseline composition is essential for distinguishing health-associated communities from those influencing pathogeneses in OM.

### 4.2. Microbiome Profiling Methods (Cultures vs. Sequencing Techniques)

The study of the middle ear microbiome has evolved considerably over the past six decades. Initially, investigations relied exclusively on culture-based techniques, which led to the classical model of OM pathogenesis dominated by *H. influenzae*, *S. pneumoniae*, and *M. catarrhalis*. While these methods remain clinically relevant for antimicrobial susceptibility testing, they underrepresent the true microbial diversity present in the middle ear [34,35].

Molecular approaches such as polymerase chain reaction (PCR) and quantitative PCR (qPCR) introduced higher sensitivity and allowed detection of fastidious organisms but remained limited to targeted bacteria. The real paradigm shift came with the adoption of culture-independent, community-wide methods such as 16S rRNA amplicon sequencing, which revealed the presence of previously underappreciated bacteria including *A. otitidis* and *T. otitidis*, as well as broader community shifts associated with health and disease conditions [29,30]. More recently, shotgun metagenomics and metatranscriptomics have provided functional insights, allowing strain-level resolution and identification of genes related to virulence, antimicrobial resistance, and biofilm formation [36].

Evidence from comparative studies highlights the gaps between methods. Culture-negative middle ear effusions frequently contain bacterial DNA and even RNA transcripts when analyzed with sequencing or RT-PCR, indicating the presence of metabolically active microbes despite apparent sterility on culture [26]. Similarly, sequencing has repeatedly revealed bacteria such as *Alloiococcus* and *Turicella*, virtually absent in culture-based studies but often abundant in chronic OM [29,30].

Thus, culture and sequencing should be seen as complementary: culture provides actionable clinical information, while sequencing broadens our ecological and mechanistic understanding of the middle ear microbiome.

Comparative overview of profiling methods is presented in Table 1.

### 4.3. Protective Role of Commensal Organisms

The composition of the middle ear and upper respiratory tract microbiome is not only determined by the presence of pathogens but also by the protective role of commensal species. Increasing evidence suggests that certain commensal bacteria act as ecological stabilizers, preventing colonization or overgrowth of otopathogens.

Several case–control studies have demonstrated that children less prone to RAOM or COME harbor higher abundances of *Corynebacterium* spp. and *Dolosigranulum pigrum* in the nasopharynx as well as *Fusobacterium nucleatum*, *Gemella sanguinis*, and *Eubacterium sulci* [33,37,38]. These organisms are considered keystone commensals: their presence is associated with a reduced risk of pathogen dominance. Mechanistically, they may protect via competitive exclusion for epithelial binding sites, production of lactic acid and other inhibitory metabolites, or modulation of mucosal immune responses [39].

For example, Lappan et al. showed that children resistant to RAOM had significantly more *Corynebacterium* spp. and *Dolosigranulum* spp. in their nasopharyngeal microbiota, whereas those with recurrent disease were enriched in *Moraxella* spp. and *Haemophilus* spp. [33]. Similarly, Xu et al. observed that a higher abundance of these commensals correlated with reduced detection of *S. pneumoniae* and *H. influenzae* in the middle ear fluid [40]. Jo et al. defined *F. nucleatum*, *G. sanguinis*, and *E. sulci* as age-specific species, whose patterns change consistently with age and can be used to predict the child’s age from the microbiome alone. *Oribacterium parvum*, *Cardiobacterium homini*, *Streptococcus salivarius*, *Veillonella parvula*, and *Neisseria mucosa* were identified as bacteria that may modulate transitions between commensal-rich structures towards a pathogen-dominated COME (high *S. pneumoniae*, *H. influenzae*) [38].

Other organisms such as *Streptococcus salivarius* have also emerged as potential protective pathobiont-inhibiting species. In vitro studies demonstrate that *S. salivarius* isolates from children can inhibit the growth of *H. influenzae*, *M. catarrhalis*, and *A. otitidis*, raising the possibility of probiotic interventions [41].

The middle ear is usually a low-biomass site in health, yet its microbial composition is shaped by the nasopharynx and adenoids, which act as reservoirs for organisms that may ascend through the ET. A diverse, commensal-rich nasopharyngeal microbiota functions as the first line of defense against OM. Although direct evidence for protective effects within the middle ear itself is scarce, maintaining this balance, by limiting antibiotic disruption, promoting breastfeeding, or even exploring probiotic supplementation, emerges as a promising strategy for preventing disease.

### 4.4. How Colonization by H. influenzae, S. pneumoniae, and M. catarrhalis Influence the Structure of the Microbiome

The microbial ecology of the middle ear is profoundly influenced by colonization with the three classical otopathogens: *H. influenzae*, *S. pneumoniae*, and *M. catarrhalis.* These bacteria usually colonize the nasopharynx during early childhood, often in the wake of viral upper respiratory tract infections that compromise mucosal integrity and alter the commensal community. The dysbiosis of the adenoid microbiome was significantly association with the COME microbiome [38]. These pathogens can then ascend via the ET into the middle ear cavity, where the immature anatomy of children and their developing immunity provide an environment susceptible to persistence [35,42].

The presence of otopathogens does more than initiate acute infection. It actively restructures the microbial community. Effusions dominated by *H. influenzae*, *S. pneumoniae*, or *M. catarrhalis* consistently show reduced bacterial diversity, reflecting the ability of these species to suppress beneficial commensals such as *Corynebacterium* and *Dolosigranulum* [29,33]. This loss of diversity undermines ecological resilience and predisposes the middle ear to recurrent or chronic disease. Furthermore, the microbial landscape changes over time: acute infections are typically characterized by high burdens of classical otopathogens, while chronic effusions often contain less well-studied bacteria such as *A. otitidis* or *T. otitidis*, which seem to flourish in the altered environment established by the initial infection [30].

Furthermore, the microbial landscape changes over time. In preschool children (2–6 years old), anatomical and functional immaturity of the ET is likely the causative agent of OM. In older children, ET length and angle approach adult configuration, ventilation improves, and adenoidal tissue gradually decreases. While a sharp decline in OM can be attributed to maturation of EF, some children remain susceptible despite structurally mature anatomy. Jo et al. showed that in children who continue to have COME beyond the preschool years (6–12-years old), disease is strongly associated with a highly abnormal, age-independent adenoid microbiota. COME samples no longer followed the age-dependent maturation seen in healthy controls but instead showed low-diversity communities rich in *S. pneumoniae* and *H. influenzae.* This study implied that a pathogen-rich adenoid microbiome may play a greater role in promoting chronic inflammation rather than ET dysfunction alone [38].

## 5. Biofilm Formation in the Middle Ear

Biofilm biology is now recognized as a cornerstone of OM research. Rather than representing an acute, self-limited infection, pediatric OM frequently evolves into a chronic condition sustained by surface-attached microbial communities that exhibit marked tolerance to antibiotics and immune-mediated clearance [43,44]. These biofilms play a central role in recurrent and persistent disease in children and are shaped by the anatomical, immunological, and ecological features of the pediatric middle ear.

A biofilm consists of microbial cells encased in a self-produced extracellular polymeric substance (EPS) composed of polysaccharides, proteins, lipids, and extracellular DNA (eDNA), which provides structural stability, enhances nutrient retention, promotes horizontal gene transfer, and protects bacteria from antimicrobial and immune pressures [43,44]. Although the classical stages of biofilm formation, attachment, microcolony formation, maturation, and dispersal, have been extensively described [45,46,47,48,49,50,51], the relevance of these stages in pediatric OM depends strongly on host factors such as ET dysfunction, viral injury to the epithelium, and immature mucociliary and immune responses [15,20,23].

In the pediatric middle ear, initial bacterial adhesion is facilitated by inflammation-induced alterations of the mucosa, including upregulation of adhesion molecules and impaired mucociliary clearance. Otopathogens such as *H. influenzae*, *S. pneumoniae*, and *M. catarrhalis* exploit these conditions to form stable EPS-embedded communities that demonstrate substantially increased tolerance to antibiotics and oxidative stress [52,53,54,55]. Biofilms in this niche also contain metabolically heterogeneous subpopulations, including dormant persister cells that survive lethal antibiotic concentrations [29,34,56,57].

A growing body of evidence indicates that biofilms in pediatric OM are rarely monospecies. Instead, polymicrobial biofilms represent the dominant in vivo state and introduce additional layers of complexity relevant to chronicity and treatment failure. Cooperative interactions between *H. influenzae* and *M. catarrhalis* enhance biofilm biomass, structural integrity, and antibiotic tolerance compared with single-species communities [43,44,52,53,55]. *M. catarrhalis* can protect *H. influenzae* from pneumococcal antagonism [58,59], while *H. influenzae* modulates pneumococcal autolysis and oxidative stress responses, stabilizing mixed biofilm survival [57]. These cross-species interactions reshape quorum sensing (QS) dynamics, alter metabolic gradients, and create protective micro-niches that promote persistence and reseeding of the middle ear even after therapy [58,60].

## 6. Influence of the Middle Ear Microbiota on Biofilm Formation and Disease Persistence

### 6.1. Interactions Between Microbiota and Pathogen Bacteria

The microbiota can be both protective against and contribute to OM by modulating inflammation and immune responses. A healthy microbiota in the upper respiratory tract competes with pathogens and activates antimicrobial and anti-inflammatory components [59,61]. Frequent commensal bacteria, *Corynebacterium* spp. and *D. pigrum*, can resist colonization of otopathogens by using several mechanisms. By direct antibiosis, *D. pigrum* alone or with *C. pseudodiphtheriticum* inhibits *Staphylococcus aureus* and *S. pneumoniae* using synthesized antagonists and/or combined metabolites that mediate killing or growth suppression [62,63]. Also, a *Corynebacterium*-mediated habitat can be modified by release of antipneumococcal free fatty acids which cause direct chemical antagonism. Nutrient competition is also a mechanism of pathogen interference. *D. pigrum* relies on host or co-colonizers for essential micronutrients, which creates a complex metabolic interdependence and competitive environment. Synergistic growth with nasal *Corynebacterium* spp. can secure or redirect resources toward a community composition that is more restrictive to *S. pneumoniae*. This results in reduced pneumococcal growth across diverse serotypes in the presence of *D. pigrum* and *C. pseudodiphtheriticum* [63]. Signaling-level interference is reflected in disrupting QS or the reception of environmental cues that trigger adhesion/biofilm genes [64]. Commensals enzymatically inactivate autoinducers, degrading N-acyl-homoserine lactones (AHLs) with lactonases, acylases, or oxidoreductases so signals never reach the activation threshold of biofilm and virulence regulons [65]. This consequently impedes coordinated bacterial group behaviors like matrix production and adhesion. Gram-positive commensals secrete non-cognate autoinducing peptides (AIPs) and the autoinducer-2 (AI-2) [66], which competitively block signaling pathogen peptide-QS, demising the expression of accessory gene regulator (*agr*) system virulence factors in vivo [67]. By disrupting the QS systems of pathogens, commensals can repress pathogen colonization and further inhibit the formation of biofilms [68].

### 6.2. Microbiota Dysbiosis in the Middle Ear

**Figure 2:**
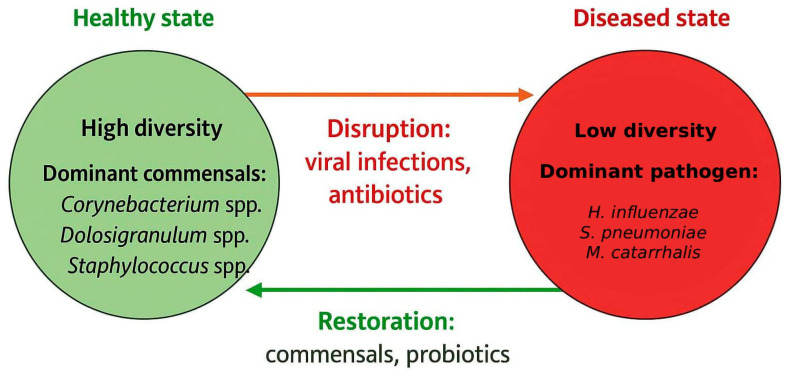
Ecological balance of middle ear microbiome.

Dysbiosis of the middle ear microbiota is a result of a shift in the components of bacterial community compared to their levels in a healthy state, and it is linked to OM [69]. This microbial imbalance is characterized by a loss of beneficial bacteria, an overgrowth of pathogen bacteria, or a decrease in overall microbial diversity, linked to changes in host–microbe interactions which promote infections and chronic inflammatory conditions [70] (Figure 2).

Multiple factors can cause dysbiosis of the middle ear microbiota, predisposing children to OM. Antibiotic exposure is a well-documented factor contributing to middle ear microbiota dysbiosis. Broad-spectrum antibiotics reduce the protective bacteria such as *Corynebacterium* spp. and *Dolosigranulum* spp. while sparing biofilm-embedded otopathogens [69]. Recurrent viral infections (RSV, influenza, adenovirus) also play an important role by impairing mucociliary clearance, altering epithelial receptor expression, and providing ground for secondary bacterial colonization. Host-related predispositions such as immature mucosal immunity, lower secretory IgA levels, and genetic variations in innate receptors can further increase this effect and impair early pathogen recognition [71]. Finally, environmental pressures including daycare attendance, passive smoke exposure, air pollution, and close sibling transmission alter microbial ecology, often reducing diversity of microbiota and enriching an otopathogen-dominant environment [72]. Pneumococcal conjugate vaccines (PCVs) vaccination could also disrupt the microbiome by decreasing vaccine-specific serotype carriage [73,74]. Higher diversity and stability of microbiota was observed in PCV13-vaccinated infants, which might be consistent with a lower susceptibility to otitis media [73,75]. The use of conjugate *H. influenzae* serotype b (Hib) vaccines have led to a significant reduction in pharyngeal carriage, decreasing invasive disease. Non-typeable *H. influenzae* (NTHi), which was not targeted by standard Hib vaccines, NTHi carriage, and NTHi-associated diseases such as otitis media, was mostly unaffected and consequently may even become relatively more prominent [76]. More recently, protein D-containing pneumococcal conjugate formulations (PHiD-CV) have been shown to induce anti-protein D responses and to reduce NTHi carriage and NTHi-positive middle ear infections [77]. By actively restructuring the nasopharyngeal microbiome, vaccination against *S. pneumoniae* and *H. influenzae* may change competitive and cooperative interactions among airway bacteria, with potential consequences for the balance between otopathogens and protective commensals.

### 6.3. Microbiota Dysbiosis and Immune Response Modulation

Microbiota colonize the mucosal barrier and settle in the respiratory tract, playing integral roles in initiating an immune response, from maintaining homeostasis to responding to infectious challenges [78]. Commensal bacteria exert protective functions against respiratory pathogens through host-mediated immunity and direct action by inhibiting/killing pathogens and competing for colonization. Mucosal defense is stimulated by activating epithelial pattern-recognition receptors and low-level immune activation, which sustains antimicrobial peptide secretion (defensins, cathelicidins), supports secretory IgA–mediated exclusion, and preserves integrity of tight junction and ciliary function [20,79]. These actions maintain a stable barrier against otopathogens. Experimental dysbiosis of airway microbiota weakens innate immunity responses in myeloid and innate-like lymphocytes (macrophage interferon responses, NK cell activity). When pathogen bacterial communities prevail, the host responds typically by shifting cytokine expression: elevated IL-1β, IL-6, TNF-α, and IL-8 sustain neutrophil recruitment, inflammation, and mucus hypersecretion [20,80]. Other innate immunity changes include altered Toll-like receptor (TLR) signaling that can be either an overly aggressive or suppressed immune reaction, depending on which microbes dominate [81].

During inflammation driven by pathogens, polymeric immunoglobulin receptor (pIgR), a key protein for mucosal immunity, can be downregulated, trapping polymeric IgA in the tissue and reducing luminal sIgA availability [82]. Reduced sIgA weakens immune exclusion and the protective commensal barrier. Also, commensal-specific T cells can become drivers of low-grade, biofilm-enabling inflammation in the middle ear by shifting from −10 secretion that suppresses pro-inflammatory responses to IL-17 secretion that promotes neutrophil-recruiting inflammation [83]. This inflammatory state disrupts epithelial junctions and impairs ciliary beating, thickens mucus, and generates eDNA and other polymers that facilitate bacterial surface attachment and biofilm formation [70]. These changes can explain how dysbiosis of the middle ear microbiota can cause persistence of biofilm-forming pathogens despite therapy and host clearance attempts.

### 6.4. The Relationship Between Microbiome Dysbiosis and Biofilm Formation

Microbiome dysbiosis in the upper airway and middle ear represents favoring opportunistic otopathogens and serves as one of the facilitating factors of biofilm formation in otitis media. Episodes of repeated upper respiratory infections, along with other environmental factors causing microbiome dysbiosis, can disrupt mucociliary clearance, damage epithelial cilia, and disrupt expression of adhesion receptors (such as ICAM-1) on epithelial cells, increasing the likelihood of bacterial attachment [84]. The middle ear experiences increased oxygen absorption across the inflamed mucosa, leading to a significant reduction in total middle ear pressure and impaired ventilation [85]. Some of the otopathogens, especially facultative anaerobes like *H. influenzae* and *M. catarrhalis*, utilize these conditions for increased mucosal adherence as an initial step in biofilm formation [86,87]. These host inflammatory responses reinforce the biofilm niche, allowing these bacteria to transition into biofilm growth modes characterized by adherence, extracellular matrix production, and metabolic adaptation.

After initial attachment to the mucosal surface, bacteria begin clustering into microcolonies. eDNA released through autolysis or bacteriophage induction provides the initial matrix scaffold [88]. Biofilm maturation involves developing a three-dimensional architecture with thick EPS which consists of extracellular DNA, proteins, lipids, and polysaccharides. In *S. pneumoniae* biofilm, capsule downregulation occurs during maturation. Non-encapsulated or low-capsule phenotypes dominate biofilm biomass which enhances aggregation and nutrient diffusion [89]. This stage is strongly influenced by QS systems, like LuxS/AI-2, which are specific for *S. pneumoniae*, *H. influenzae*, and *M. catarrhalis* and coordinate biofilm thickness, virulence factor production, and resistance phenotypes [90,91,92].

Within mature biofilms, bacteria undergo phenotypic changes caused by stress responses, QS signals, and nutrient limitation within the biofilm microenvironment. These changes involve entering a slow-growth metabolic state, accompanied by increased genetic competence and horizontal gene transfer that enhance bacterial adaptation and antibiotic resistance [93]. Within mature pneumococcal biofilms, some bacteria differentiate to dormant persister cells, which are highly tolerant to antibiotics, oxidative stress, and the host’s immune response [94]. Dysbiosis and biofilm maturation create a self-reinforcing cycle, where microbial imbalance facilitates biofilm formation and biofilms maintain dysbiosis by protecting pathogens and preventing commensal recovery.

There is strong evidence that some otopathogens cooperate and interact with each other within the biofilm formation, causing more severe and persistent infections and higher burden of antibiotic use. Dysbiotic states in which *H. influenzae* and *S. pneumoniae* are dominant together favor cooperative biofilm formation for both species. *H. influenzae* may inhibit pneumococcal autolysis and stabilize mixed communities [95]. Also, changes in pH, oxygen, and nutrient availability can influence pneumococcus to reduce its competitive pressure, which allows *H. influenzae* to persist [96]. Change in virulence-associated gene expression (adhesins, immune modulators) can happen in both species. *H. influenzae* adapts to the presence of pneumococcus by upregulating oxidative stress defenses and biofilm-associated genes (catalases, peroxidases, DNA repair enzymes that neutralize hydrogen peroxide), reducing susceptibility to pneumococcal antagonism and stabilizing its persistence within mixed biofilms [97].

**Figure 3:**
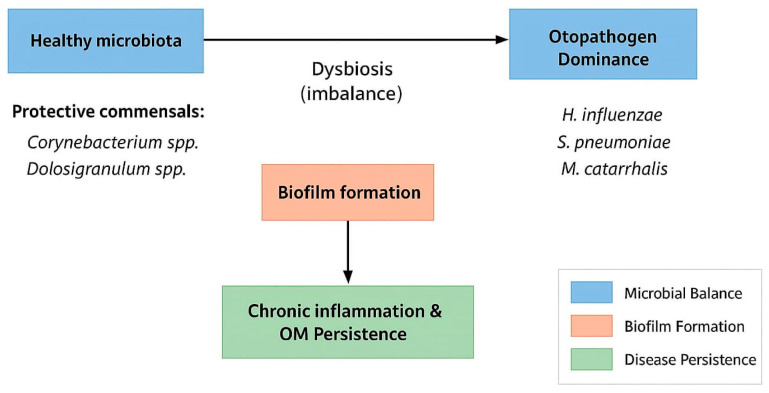
Microbiota dysbiosis and biofilm formation in otitis media.

Colonization with *M. catarrhalis* plays a key role in stabilizing mixed-species communities in the nasopharynx and middle ear by promoting the persistence of other otopathogens. Experimental models have shown that *M. catarrhalis* protects *H. influenzae* from the bactericidal activity of *S. pneumoniae* in a polymicrobial biofilm [49]. This stabilizing effect is not fully explained by catalase activity alone, suggesting additional protective mechanisms such as metabolic cooperation, quorum signaling modulation, and physical incorporation into the biofilm matrix. The presence of *M. catarrhalis* enhances both biofilm biomass and antibiotic tolerance, as well as long-term colonization by *H. influenzae* and *S. pneumoniae*. It enables cooperative dynamics among otopathogens, which could explain why carriers of *M. catarrhalis* are more prone to recurrent or chronic otitis media [98]. These findings suggest that OM should be viewed less as an infection but as a microbiome dysbiosis-based disorder, in which the balance between commensals and otopathogens determines the clinical outcome of the disease (Figure 3).

## 7. Current Research and Future Directions in Microbiome- and Biofilm-Targeted Therapy

### 7.1. Microbiome-Targeted Therapy

Microbiome-targeted therapies for OM are increasingly under investigation; many of the available strategies are not adapted for treatment of this disease (Table 2). One promising direction involves live biotherapeutic products (LBPs), pharmaceutical-grade probiotics created to modulate the respiratory microbiome by targeted competitive inhibition of otopathogens or immune modulation. For example, intranasal application of *Haemophilus haemolyticus* has shown potential in inhibiting NTHi colonization, while nasal sprays containing *Streptococcus salivarius* and *Streptococcus* strains have demonstrated reductions in acute and secretory OM, even reducing the need for surgical intervention [41,99].

The development of next-generation probiotics is rapidly evolving beyond traditional, broad-spectrum strains toward live, strain-specific engineered microorganisms designed for a precise therapeutic effect [41]. These genetically modified microbes can be engineered to produce anti-inflammatory molecules, antimicrobial peptides, or quorum sensing inhibitors, directly targeting pathogenic bacteria or modulating host immune responses to reduce inflammation and biofilm formation. Advances in site-specific delivery systems (nasal sprays, hydrogels, and resorbable films) are improving the precision of probiotic administration by enabling targeted colonization and prolonged retention in the nasopharynx or middle ear [100]. These delivery modalities enhance the survival and activity of probiotics in anatomically challenging sites, optimizing their therapeutic impact [101]. The co-administration of synbiotics, combining selected probiotic strains with prebiotics that selectively sustain beneficial bacteria, represents a promising strategy to restore and maintain a healthy microbiome [102]. By promoting synergistic interactions within the microbiome, synbiotics help maintain the resilience and functional stability of microbial communities, providing protection against otopathogen overgrowth and the development of chronic middle ear disease.

Postbiotics, or non-living microbial derivatives, remain an attractive concept for modulating local immunity or microbial networks, though data in the OM context are currently sparse [103].

Using bacteriophages, as phage therapy, to target specific pathogens demonstrates theoretical benefits in penetrating biofilms, and preliminary studies suggest that trans-tympanic delivery could overcome the tympanic membrane barrier, though no clinical applications in acute OM have yet been conducted [104].

Microbiota transplants, such as fecal microbiota transplantation (FMT), have been previously established in the gastrointestinal environment, but the concept remains experimental for otitis media. Their applicability is limited by delivery routes and specific microbiome matching [105]. Two recent studies have published data on nasal microbiota transplantation (NMT) in treatment of chronic rhinosinusitis, with possible implications for countering antimicrobial resistance in respiratory infections [106].

Use of engineered microbes, genetically programmed to produce therapeutic molecules or inhibit pathogens, is still a theoretical strategy. There are is vitro research proving that this engineered strain of *Mycoplasma pneumoniae* was very efficient against an acute *Pseudomonas aeruginosa* lung infection [107]. Given that *P. aeruginosa* is one of the frequently isolated pathogens in CSOM, research in this direction could be promising. Across all these approaches, they face common challenges: establishing stringent regulatory pathways for living therapeutics, ensuring safety and effective delivery modalities, managing microbial strain specificity and colonization stability, and potential immune reactions or unintended ecological disruptions [108].

The future of treating OM is shifting toward precision microbiome modulation, individualized microbiome-specific interventions that aim to restore microbial balance rather than eradicate bacteria without discrimination. The goal is to create treatments based on a patient’s unique nasopharyngeal or middle ear microbiome composition, which is now accessible through advanced microbial profiling technologies such as 16S rRNA sequencing and whole metagenomic shotgun sequencing [13,109,110]. These tools enable the identification of diagnostic microbiome signatures, allowing for patient stratification and prediction of disease risk or treatment responsiveness [111]. This proved especially relevant in pediatric OM, where microbial heterogeneity influences both pathogenesis and therapeutic outcomes.

**Table 2:** Microbiome-targeted therapy.

Therapeutic Strategy	Key Findings	Challenges
LBPs (nasal probiotics)	*H. haemolyticus* and *S. salivarius*/*S. oralis* strains reduce OM episodes and surgical need (intranasal use)	Complex host–microbe interactions, variability in patient responses, delivering issues, regulatory guidelines [112]
Next-generation Probiotics	Can be designed with the ability to produce specific beneficial molecules, modulating immune responses; more targeted and effective therapeutic approach	Need for expensive, anaerobic culture media for industrial-scale production; need for effective delivery systems to ensure viability, safety concerns, especially in vulnerable populations [113]
Microbiota Transplants	Widely used in gut disorders; limited, experimental application in OM	Delivery routes, specific microbiome matching, donor selection, screening to prevent pathogen transmission, lack of standardized protocols and regulatory guidelines [114]
Postbiotics	Conceptually promising; little OM-specific data currently	Lack of a standardized definition, regulatory guidelines, delivery issues and absorption, more data on safety and dosing [115]
Phage Therapy	Potential for biofilm penetration; trans-tympanic delivery explored; no clinical OM use yet	Bacterial resistance to phages, limited host specificity of phages, delivery issues, unpredictable pharmacokinetics and pharmacodynamics, potential immune system interactions, and regulatory guidelines [116]
Engineered Microbes	Conceptual therapeutic potential; largely preclinical	Limited survival in targeted environment, potential off-target effects on the respiratory microbiota, delivering issues, high genetic diversity and rapid evolution of pathogens, safety issues [117]
Precision microbiome modulation	Alteration of molecular signaling, immune modulation, and drug metabolism of microbial communities for personalized health outcomes	Microbiome’s complexity, difficulties in achieving stable and predictable modulation, limitations of tools for clinical application [118]

### 7.2. Antibiofilm Agents

Biofilm-targeted therapeutics aim to inhibit or eradicate biofilm communities responsible for chronic infections (Table 3). Among the most promising candidates are biofilm-disrupting agents like N-acetylcysteine (NAC), DNase, and dispersin B, which degrade major biofilm matrix components and weaken the EPS [119,120,121,122]. Another interesting approach involves QS inhibitors (QSIs). These agents, both natural and synthetic molecules, have shown efficacy in reducing biofilm formation and enhancing antibiotic susceptibility across various pathogens [123,124].

Recent advances in biofilm therapeutics focus on overcoming the intrinsic resistance of biofilm communities through smart drug delivery systems. Nanoparticles and liposomes engineered to penetrate the dense EPS can deliver antibiotics or biofilm-degrading enzymes directly [125]. These delivery systems are often designed to respond to specific environmental conditions, triggering a controlled release and minimizing off-target effects [126]. Antibiotics in biofilm-specific formulations use carriers like liposomes, hydrogels, or nanoparticles to overcome restricted antibiotic penetration, altered bacterial metabolism, and increased resistance. These specialized formulations aim to improve antibiotic delivery, protect antibiotics from degrading enzymes in the biofilm, or utilize compounds that disrupt the biofilm matrix [127].

Bacteriophages are increasingly explored for their ability to target bacteria embedded within biofilms, offering adjunctive or alternative therapeutic pathways, but their clinical use remains limited [128].

Anti-adhesives are a novel strategy for preventing bacterial infections and biofilm formation by blocking initial bacterial attachment. Compounds that show promise for future research in treatment of otitis media biofilm include natural substances like probiotics (Lactobacillus), mannosides (C-mannosides), and hyaluronic acid [129,130,131,132,133].

Pilicides and curlicides are anti-biofilm agents that inhibit the formation of bacterial surface protein fibers, pili and curli, crucial for attachment to host surfaces and to each other as a first step in biofilm formation. They have been studied in the context of urinary tract infections (UTIs) caused by *Escherichia coli* and other general biofilm infections, but their specific efficacy in the treatment of otitis media (ear infection) biofilms is still in the research phase [134].

**Table 3:** Anti-biofilm therapy.

Therapeutic Strategy	Effect	Challenges
Biofilm-disrupting agents	Dispersin B degrades poly-β-1,6-N-acetyl-D-glucosamine (PNAG), disrupting biofilm matrix; DNase degrades extracellular DNA (eDNA); NAC disrupt the extracellular polymeric substance (EPS) matrix by breaking disulfide bonds; direct antimicrobial effect, increased oxidative stress	Variable effect on mature biofilms, potential shielding of target matrix components
Quorum sensing inhibitors	Natural and synthetic QSIs suppress biofilm formation, reduce virulence, and improve antibiotic response	Direct resistance, lack of specificity, limited monotherapy efficacy, wash-away effect
Smart drug delivery systems (nanoparticles)	Mesoporous silica NPs (±lectin or gold layer) enable targeted antibiotic release, photothermal disruption, and enhanced biofilm penetration	Health and environmental toxicity, complex manufacturing, safety concerns
Bacteriophages	Bacterial lysis and the destruction of the biofilm structure, production of depolymerase enzymes that degrade the biofilm’s EPS	Narrow host range, bacterial resistance, storage and stability issues [135]
Antiadhesives	Creation of an overlayer on mucosal surface and prevention of bacterial adhesion	Biocompatibility, routes of application and fixation, degradation
Pilicides and curlicides	Binding to the periplasmic chaperone protein and/or chaperone-like proteins, blocking the transfer of the pilin subunit and halting the assembly of functional pili on the bacterial surface which leads to loss of ability to adhere to surfaces, colonize host tissues, and form biofilms	Questionable success metrics, need for combination therapy due to multiple bacterial virulence factors, potential bacterial resistance or evasion [136]

## 8. Conclusions and Future Perspectives

Pediatric OM remains among the most prevalent diseases of early childhood, reflecting a convergence of developmental anatomy, immunological immaturity, and the complex microbial ecology of the upper airway. The contemporary view moves beyond the outdated model of a sterile middle ear invaded episodically by pathogens. Instead, the middle ear constitutes an ecological niche shaped by dynamic interactions between the nasopharyngeal microbial reservoir, local commensals, and host defenses. Within this niche, biofilm formation has emerged as a central pathogenic mechanism, enabling microorganisms to persist, evade immune clearance, and resist conventional antimicrobial treatment. By transforming acute into recurrent and chronic inflammatory infections, biofilms account for much of the morbidity, treatment failure, and long-term sequelae associated with otitis media in children.

This reconceptualization has direct therapeutic implications. Conventional antibiotic regimens, although clinically indispensable, are insufficient against biofilm-embedded bacteria and may disrupt protective commensal populations that contribute to colonization resistance. Future interventions will increasingly rely on microbiome-based and antibiofilm strategies that restore ecological balance rather than simply eliminate pathogens.

In summary, pediatric otitis media represents a multifactorial condition shaped by host developmental factors, immune maturation, and microbiome dynamics. Understanding these complex interactions provides the foundation for more effective prevention and treatment strategies.

Viewed through this ecological and mechanistic lens, pediatric otitis media emerges not simply as an infectious disease but as the consequence of disrupted interactions between host and microbiota within a biofilm-dominated environment. Future progress in prevention and treatment will depend on translating these insights into clinically actionable strategies capable of reducing both the immediate burden of recurrent infections and the long-term risk of auditory and developmental sequelae in children worldwide.

## Figures and Tables

**Table 1:** Comparative overview of profiling methods.

Method	What Is Detected	Strengths	Limitations
Culture (aerobic/anaerobic)	Viable, cultivable bacteria	Enables antimicrobial susceptibility testing; precise species ID	Misses fastidious/slow-growing bacteria; underestimates diversity
PCR/qPCR	DNA or RNA of specific targets	High sensitivity; detects unculturable organisms	Limited to known targets; no community view; false positives possible
16S rRNA sequencing	Broad bacterial community (genus-level, sometimes species-level)	Comprehensive overview; diversity metrics; unculturable bacteria detected	Limited resolution; relative abundance only; contamination risk in low-biomass samples
Shotgun metagenomics/metatranscriptomics	DNA (genomes) or RNA (active transcripts)	Strain-level resolution; functional profiling (virulence, resistance genes)	Expensive; requires high biomass; computationally demanding

## Data Availability

No new data were created or analyzed in this study. Data sharing is not applicable.

## Abbreviations

The following abbreviations are used in this manuscript:

ET	Eustachian tube
RAOM	Recurrent acute otitis media
MEE	Middle ear effusions
RSV	Respiratory syncytial virus
COME	Chronic otitis media effusion
QS	Quorum sensing
NTHi	Nontypeable *H. influenzae*
OT	Otits media
TLR	Toll-like receptor
eDNA	Extracellular DNA
EPS	Extracellular polymeric substance
LBPs	Live biotherapeutic products
